# Blended and Technology-Enhanced Education in Pediatric Emergency Nursing: A Systematic Review

**DOI:** 10.3390/pediatric18030080

**Published:** 2026-06-11

**Authors:** Rita Nocerino, Giorgia Cerase, Emma Montella, Albina Simeoli

**Affiliations:** 1 Department of Translational Medical Science, University of Naples “Federico II”, 80131 Naples, Italy; 2 Departmental Area of Health Services, Unit of Health Services Organization, University Hospital Federico II, 80131 Naples, Italy; emma.montella@unina.it (E.M.); albina.simeoli@unina.it (A.S.); 3 Department of Emergency Care, Santobono-Pausilipon Children’s Hospital, 80129 Naples, Italy; giorgiacerase98@libero.it

**Keywords:** clinical skills, educational interventions, nursing education, pediatric nursing, professional competence, simulation training

## Abstract

Background: Pediatric emergencies are high-risk clinical situations requiring timely, accurate, and coordinated interventions. Nurses play a pivotal role in early recognition and management of acute pediatric conditions; however, the rarity and complexity of these events often limit clinical exposure and preparedness. Continuous professional education is therefore essential to ensure patient safety and high-quality care. Objective: This systematic review aimed to synthesize evidence on innovative continuing education strategies for nurses involved in pediatric emergency care, with a primary focus on studies evaluating educational effectiveness and a secondary contextual focus on studies describing training needs, perceived barriers, preparedness, and implementation conditions. Methods: The review was conducted according to PRISMA guidelines. The protocol was registered in PROSPERO (ID CRD420251120993). A comprehensive search of PubMed, CINAHL Complete, Scopus, and the Cochrane Library identified studies published between 2015 and August 2025. Primary intervention studies were used to assess educational effectiveness, whereas descriptive, observational, qualitative, and review-based evidence was retained as contextual evidence. Methodological quality was assessed using Joanna Briggs Institute (JBI) tools. Results: Forty-nine studies met the inclusion criteria, including randomized controlled trials, quasi-experimental studies, observational and cohort studies, and integrative or narrative reviews. Educational interventions mainly involved simulation-based training, blended learning, telesimulation, digital education, and structured training programs. Intervention studies suggested improvements in knowledge, technical skills, self-efficacy, and team performance, while contextual studies highlighted training needs, perceived barriers, preparedness, and implementation challenges. However, the evidence was limited by methodological heterogeneity, frequent reliance on self-reported outcomes, and limited long-term follow-up. Conclusions: Simulation-based, blended, and telesimulation-based educational strategies may be associated with short-term improvements in nurses’ preparedness and educational outcomes in pediatric emergency care. However, conclusions regarding effectiveness should be interpreted cautiously because of methodological heterogeneity, reliance on subjective outcomes, and limited evidence on long-term clinical and patient-safety outcomes.

## 1. Introduction

Emergency care involves clinical situations in which patients are at imminent risk of life and require immediate, accurate, and coordinated interventions. Critical care settings are characterized by high clinical intensity, where rapid decision-making and effective actions are essential to preserve vital functions in patients with severe physiological instability. When not promptly addressed, such instability may compromise short- or medium-term survival, requiring continuous, highly specialized care supported by constant monitoring and targeted invasive interventions aimed at stabilization [1].

In pediatric populations, emergency care presents additional challenges related to anatomical, physiological, and pathophysiological differences across age groups. Children exhibit variable clinical responses to treatments, increased vulnerability, and limited physiological reserves, making pediatric emergency management fundamentally different from adult care. Emergency situations in pediatrics therefore require age-specific assessment, rapid recognition of clinical deterioration, and timely interventions tailored to developmental characteristics and underlying pathophysiology [2].

Acute pediatric conditions such as cardiopulmonary arrest, respiratory failure, seizures, and neurological emergencies demand prompt, precise, and multidisciplinary responses. Even minor delays or errors may result in significant morbidity or mortality. In this context, nurses play a pivotal role in early assessment, triage, and implementation of life-saving interventions in accordance with international guidelines, including Pediatric Basic Life Support protocols. However, the relative rarity of pediatric emergencies, particularly in non-specialized settings, may limit nurses’ clinical exposure and preparedness, underscoring the critical importance of continuous professional education [1,3,4].

The World Health Organization has highlighted emergency and trauma care as essential strategies for reducing global morbidity and mortality, particularly in resource-limited settings, where training programs are key determinants of care quality and patient outcomes. Despite this recognition, structured and targeted educational pathways specifically addressing pediatric emergencies remain limited, identifying a priority area for healthcare education and workforce development [5].

Traditional educational approaches based on lectures and passive learning are increasingly considered insufficient to prepare healthcare professionals for the complexity and emotional demands of pediatric emergencies. In recent years, innovative educational strategies—such as simulation-based training, in situ simulation, team training, blended learning, and digital or virtual modalities—have been introduced to enhance experiential learning, technical performance, and non-technical skills, including communication, leadership, and interprofessional collaboration [6,7,8,9,10,11,12]. Simulation-based education, in particular, allows healthcare professionals to practice high-risk, low-frequency scenarios in a safe environment, improving confidence, self-efficacy, and readiness without compromising patient safety [9,10,12].

Despite growing interest and encouraging preliminary findings, the existing literature presents significant limitations. Most studies focus primarily on short-term educational outcomes, rely on self-reported measures, or adopt heterogeneous methodological designs. Robust evidence on the long-term impact of innovative educational strategies on clinical practice, patient outcomes, and nurses’ psychological well-being—including burnout reduction—remains scarce [3,13]. Moreover, evidence regarding the effectiveness of emerging technologies such as virtual reality in pediatric emergency training is still limited, with few studies providing realistic physiological simulations or comprehensive evaluations of both professional competencies and clinical outcomes [14].

In light of these gaps, the present systematic review aims to critically evaluate the effectiveness of innovative continuing education strategies for nurses involved in pediatric emergency care. By synthesizing available evidence, this review seeks to support the implementation of evidence-based, sustainable, and transferable educational models capable of enhancing nurses’ technical, cognitive, and relational competencies, ultimately contributing to improved patient safety and quality of pediatric emergency care.

## 2. Methods

### 2.1. Study Design and Reporting Standards

This systematic review was conducted in accordance with the Preferred Reporting Items for Systematic Reviews and Meta-Analyses (PRISMA) guidelines (see Supplementary Materials: PRISMA 2020 Checklist). The review protocol was prospectively registered in the International Prospective Register of Systematic Reviews (PROSPERO; ID CRD420251120993).

### 2.2. Eligibility Criteria

Studies were selected based on predefined eligibility criteria structured according to population, intervention, comparison, and study characteristics.

Eligible studies included qualified nurses involved in the management of pediatric emergencies and addressing basic, post-basic, or continuing professional education. Consistent with the refined scope of the review, studies focusing exclusively on undergraduate or pre-licensure nursing education were excluded.

Interventions of interest consisted of advanced educational tools and innovative training methodologies, including but not limited to simulation-based education, digital learning, blended learning, telesimulation, and other structured continuing professional development programs.

The comparator was defined as the absence of a specific educational intervention targeting nurses involved in pediatric emergency care or the use of traditional training approaches without innovative components.

Studies of any methodological design were considered eligible. Included studies focused on pediatric emergency care involving children and adolescents aged 0–18 years.

For feasibility and accuracy of full-text assessment, data extraction, and methodological appraisal, articles published in English or Italian between 2015 and 2025 were included.

Exclusion criteria comprised studies not specifically addressing pediatric emergencies, publications in languages other than English or Italian, studies published outside the selected time frame, and duplicate, incomplete, or insufficiently reported studies.

For the purpose of synthesis, included studies were categorized according to their contribution to the review question. Studies evaluating a defined educational intervention were considered effectiveness studies and were used to assess the impact of educational strategies on knowledge, skills, self-efficacy, performance, or clinical/process outcomes. Descriptive, qualitative, and observational studies that did not evaluate a specific educational intervention were retained as contextual evidence only to describe educational needs, perceived barriers, preparedness, confidence, and implementation-related factors. These studies were not used to draw conclusions regarding intervention effectiveness.

The original PROSPERO registration included a broader educational scope encompassing both undergraduate and post-graduate nursing education. During the review process, the eligibility criteria were refined to focus specifically on continuing professional education, basic/post-basic training, and professional development among qualified nurses involved in pediatric emergency care. Accordingly, studies focused exclusively on undergraduate or pre-licensure nursing students were excluded. This refinement was introduced to improve conceptual coherence and applicability to clinical practice, as pre-licensure education differs substantially from continuing education for practicing nurses in terms of learners’ clinical responsibilities, prior experience, educational objectives, and expected transferability to real-world pediatric emergency settings.

### 2.3. Information Sources and Search Strategy

A comprehensive literature search was conducted across four major electronic databases: PubMed, CINAHL Complete, Scopus, and the Cochrane Library. The search aimed to identify studies evaluating the effectiveness of continuing education strategies for nurses involved in pediatric emergency care, with a focus on innovative educational tools and methodologies.

The search covered publications from January 2015 to August 2025 to ensure the inclusion of recent and methodologically relevant evidence. The final search was conducted in August 2025. Search terms included combinations of keywords related to nursing education, pediatric emergency care, and educational effectiveness. The same core keyword-based search strategy was applied across the four databases and adapted only to the specific database interface when required. The search strategy was therefore primarily keyword-based. Although this approach ensured consistency and reproducibility across databases, no controlled vocabulary terms were incorporated. The complete search strategy for each database is reported in Table 1.

### 2.4. Study Selection Process

The database search identified a total of 640 records (PubMed n = 137; CINAHL n = 182; Scopus n = 317; Cochrane Library n = 4). All records were imported into web-based Rayyan software for screening [15].

After removal of 38 duplicates, 602 records underwent title and abstract screening.

**Figure 1:**
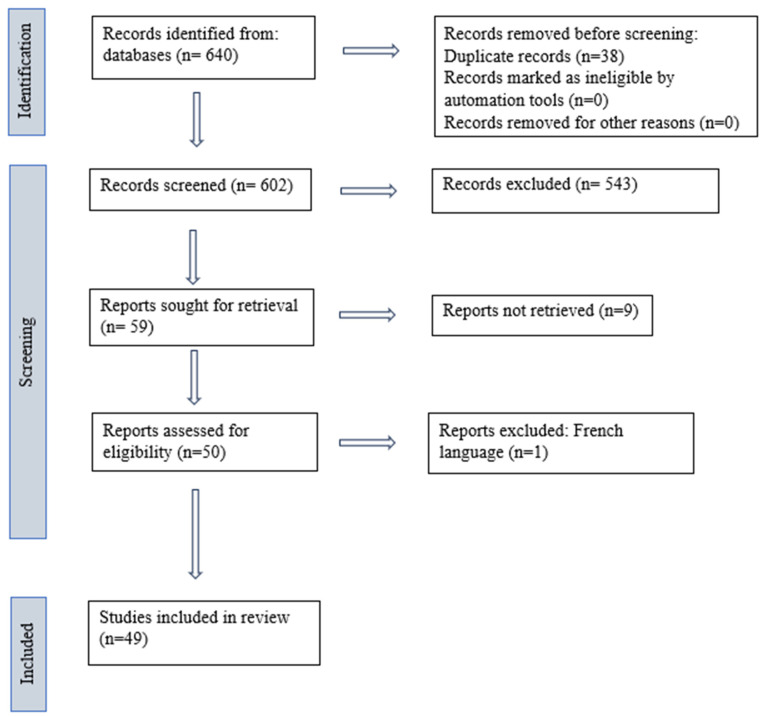
PRISMA flow diagram.

The study selection process is illustrated in the PRISMA 2020 flow diagram (Figure 1).

Two reviewers (RN and GC) independently screened all titles and abstracts in a blinded manner. Studies that did not meet the inclusion criteria were excluded. Any disagreements were resolved through discussion and consensus during joint review meetings. Full-text articles were subsequently assessed for eligibility, and discrepancies were again resolved by consensus.

For reports not immediately available through database links, full-text retrieval was attempted through institutional subscriptions, publisher websites, DOI- and title-based searches, Google Scholar, and library holdings. Reports that could not be obtained after these attempts were classified as not retrieved.

### 2.5. Data Extraction

Data were independently extracted by two reviewers using a standardized data extraction form developed for this review. The extracted variables included author, year of publication, country, study design, clinical setting, participant characteristics, type and duration of the educational intervention, comparator or control condition when applicable, outcome measures, timing of assessment, follow-up duration, main findings, and study limitations. Disagreements were resolved through discussion and consensus among the review team.

### 2.6. Risk of Bias Assessment

Although the original PROSPERO protocol planned the use of RoB-2 and QUADAS tools, the final evidence base included heterogeneous study designs for which these instruments were not uniformly applicable. RoB-2 is specifically designed for randomized trials, whereas QUADAS is intended for diagnostic accuracy studies; therefore, these tools were not appropriate for the full range of included designs. The appraisal strategy was therefore refined, and Joanna Briggs Institute (JBI) critical appraisal tools were used according to study design to ensure a more appropriate and consistent methodological assessment (Table 2).

The following checklists were applied: the JBI Critical Appraisal Checklist for Randomized Controlled Trials for randomized studies [16,17,18]; the JBI Checklist for Quasi-Experimental Studies for pre–post designs with or without control groups [3,4,5,9,13,19,20,21,22,23,24,25,26,27,28,29,30,31,32,33,34,35,36,37]; the JBI Checklist for Analytical Cross-Sectional Studies for survey and correlational studies [6,38,39,40,41,42,43,44,45,46]; the JBI Checklist for Case Series and Cohort Studies for longitudinal or descriptive studies [47,48,49,50,51,52,53]; and the JBI Critical Appraisal Checklist for Systematic Reviews and Research Syntheses for integrative reviews [54,55,56].

Two reviewers independently assessed each study, with discrepancies resolved through discussion. Key domains evaluated included randomization and allocation procedures, baseline comparability of groups, validity and reliability of outcome measures, control of confounding factors, completeness of data, blinding of participants and assessors, and appropriateness of statistical analyses. Overall risk of bias was categorized as low, moderate, or high based on the number and severity of identified methodological concerns.

### 2.7. Data Synthesis and Methodological Considerations

Although the primary focus of the review was on studies evaluating educational interventions, integrative and narrative reviews identified through the search were retained only as secondary contextual evidence. These reviews were not included in the subgrouped synthesis of primary intervention studies, were not used to support effectiveness claims, and were not combined with primary studies when interpreting educational outcomes. Their inclusion was intended to provide contextual information on broader educational themes, training needs, implementation challenges, and gaps in the literature. This approach represents a methodological refinement from the original PROSPERO protocol and was introduced to support the interpretation of a heterogeneous evidence base while maintaining a clear distinction between primary effectiveness evidence and secondary contextual evidence.

As anticipated in the PROSPERO protocol, a quantitative meta-analysis was considered only in the presence of sufficient homogeneity across studies. After data extraction, heterogeneity was assessed qualitatively by comparing study designs, participant populations, clinical settings, educational intervention characteristics, comparators, outcome domains, measurement instruments, timing of outcome assessment, and availability of extractable effect estimates. This assessment showed substantial clinical, methodological, and outcome heterogeneity. The included studies differed markedly in design, ranging from randomized controlled trials and quasi-experimental studies to observational, qualitative, implementation, quality improvement, and review-based evidence. Interventions also varied in content, duration, modality, intensity, and delivery format, while outcomes were measured using heterogeneous tools, time points, and reporting formats.

Because no sufficiently homogeneous subset of primary intervention studies reported comparable outcomes using compatible measures and extractable effect estimates, quantitative pooling was not considered appropriate. Accordingly, statistical heterogeneity measures, such as I^2^, were not calculated, and a narrative synthesis approach was adopted. To enhance interpretability despite heterogeneity, findings from primary intervention studies were narratively grouped according to educational outcome domains and clinical settings. Outcome domains included knowledge acquisition, technical skill performance, non-technical skills/self-efficacy, and patient safety or process-related outcomes. Clinical settings were grouped, where possible, into specialized pediatric settings, such as pediatric intensive care units, neonatal intensive care units, tertiary pediatric hospitals, and pediatric emergency departments, and general, community, or resource-limited emergency settings. Evidence from primary intervention studies was synthesized to evaluate educational effectiveness, whereas descriptive, observational, qualitative, and review-based evidence was used only to contextualize training needs, perceived barriers, preparedness, and implementation-related factors.

Methodological quality across studies was generally moderate, with stronger internal validity observed in more structured experimental designs. Randomized controlled trials showed moderate risk of bias, mainly due to the inability to blind participants and the use of perceptual outcomes. Quasi-experimental and cross-sectional studies frequently exhibited moderate-to-high risk of bias related to lack of control groups, reliance on self-reported measures, and limited generalizability. Cohort and implementation studies demonstrated moderate risk of bias, while integrative and narrative reviews showed moderate quality, primarily due to heterogeneity and lack of standardized reporting.

## 3. Results

### 3.1. Study Selection

The database search identified a total of 640 records. After removal of 38 duplicates, 602 records were screened based on titles and abstracts. Of these, 543 records were excluded because they did not meet the eligibility criteria. Fifty-nine reports were sought for retrieval, of which 9 could not be retrieved despite additional retrieval attempts. Therefore, 50 full-text reports were assessed for eligibility. One report was excluded because it was published in French. Ultimately, 49 studies met the inclusion criteria and were included in the systematic review.

### 3.2. General Characteristics of Included Studies

A total of forty-nine studies published between 2015 and 2025 met the inclusion criteria. The included studies employed a variety of methodological designs, including randomized controlled trials, quasi-experimental studies, cross-sectional surveys, cohort and implementation studies, as well as integrative and narrative reviews, as detailed in Table 3. The integrative and narrative reviews were considered secondary contextual evidence and were not used to draw conclusions regarding the effectiveness of educational interventions. Accordingly, they were excluded from the subgrouped synthesis of intervention-effectiveness findings and were used only to contextualize educational needs, implementation issues, and literature gaps. Most studies were conducted in hospital settings, particularly pediatric emergency departments, pediatric intensive care units, and acute pediatric wards, with a smaller number carried out in community hospitals or academic centers [21,32].

The majority of interventions targeted pediatric or emergency nurses specifically, while a limited number of studies adopted an interprofessional approach involving physicians, pharmacists, or other healthcare professionals, with nurses remaining the primary recipients of the educational interventions [13,34]. Sample sizes varied widely, ranging from small pilot studies with fewer than 30 participants to large multicenter investigations involving more than 400 nurses [38].

Geographically, the studies were distributed across North America, Europe, Asia, and South America, providing heterogeneous perspectives on educational strategies and their applicability within different healthcare systems [31,43].

### 3.3. Effectiveness Evidence from Primary Intervention Studies

The following subsections summarize findings derived from primary studies evaluating defined educational interventions. These findings were used to interpret educational effectiveness in relation to intervention type, outcome domain, and clinical setting. Descriptive, qualitative, observational, and review-based studies were not included in this effectiveness synthesis and are presented separately as contextual evidence.

### 3.4. Educational Interventions Evaluated in Primary Studies

Simulation-based education emerged as the most frequently adopted training strategy. Interventions included high-fidelity simulation scenarios, low-cost simulation workshops, and hybrid models combining practical exercises with theoretical instruction [13,32,33]. These programs aimed to balance flexibility with experiential learning and were reported to support the reinforcement of core competencies required in pediatric emergency care.

Blended learning approaches were also commonly reported, integrating face-to-face teaching with digital resources, interactive modules, and structured feedback sessions to reinforce both theoretical knowledge and practical skills [4,53]. These programs aimed to balance flexibility with experiential learning and were particularly effective in reinforcing core competencies required in pediatric emergency care.

E-learning and telesimulation gained prominence, especially in response to the COVID-19 pandemic. These interventions included asynchronous online modules, interactive decision-making platforms, podcasts, and real-time remote simulation sessions designed to replicate pediatric emergency scenarios in virtual environments [21,35]. The flexibility of these formats facilitated broader participation, including healthcare professionals from geographically remote or resource-limited settings.

Only one included study explicitly evaluated a virtual reality (VR)-based intervention, consisting of a neonatal emergency simulation program for NICU nurses [14]. This study reported improvements in OSCE performance, knowledge retention, decision accuracy, stabilization time, and safety-related outcomes. However, because VR was represented by a single study, these findings should be interpreted as preliminary.

Traditional in-person educational approaches, such as workshops, lectures, and internal training sessions, were still employed in several studies.

Although less innovative, these interventions were associated with improvements in baseline knowledge and standard clinical practices among pediatric nurses.

Particularly when addressing fundamental procedures and documentation processes [34,53].

### 3.5. Effectiveness Outcomes in Primary Intervention Studies

Outcome measures across studies focused on four main domains: knowledge acquisition, technical skill performance, patient safety–related outcomes, and self-efficacy or professional confidence.

Several intervention studies reported short-term improvements in post-training knowledge scores compared with baseline assessments [33,35]. Simulation-based interventions, both in-person and telesimulated, were associated with notable improvements in technical performance, including adherence to procedural checklists and execution of critical manoeuvres such as cardiopulmonary resuscitation, airway suctioning, and trauma management [4,21,32].

Although less frequently evaluated, patient safety-related outcomes indicated a reduction in clinical errors following educational interventions. For example, training programs focused on medication reconciliation in pediatric emergency departments resulted in fewer therapeutic discrepancies [53].

Several primary intervention studies also reported improvements in self-efficacy and professional confidence. Nurses described increased readiness, role clarity, and confidence when managing critical pediatric situations after participation in simulation-based, telesimulation, or structured educational programs [13,21,35]. These findings suggest that educational strategies not only enhance measurable knowledge and skills but also strengthen nurses’ professional self-perception in high-acuity pediatric settings. Given the heterogeneity of interventions, outcomes, and settings, findings were further organized into narrative subgroups to provide a more granular interpretation of the evidence.

### 3.6. Subgrouped Synthesis of Intervention-Effectiveness Evidence

To provide more specific insights despite heterogeneity, findings from primary intervention studies were narratively grouped according to educational outcome domains and clinical settings. With regard to knowledge acquisition, several structured educational interventions, including traditional teaching, online modules, blended learning, simulation-based approaches, and structured clinical training programs, reported short-term improvements in post-training knowledge scores. These gains were reported in studies addressing pediatric emergency preparedness, tracheostomy care, fever and febrile seizures, neurological or level-of-consciousness assessment, telesimulation-based emergency training, and other pediatric care procedures [5,6,24,25,29,33,35,51].

Technical skill outcomes were most frequently assessed in simulation-based, in situ simulation, telesimulation, and procedure-focused programs. These outcomes included adherence to procedural checklists, cardiopulmonary resuscitation performance, airway management, trauma-related actions, peripheral or central venous access, ultrasound-guided vascular access, and other emergency or procedural skills [4,9,27,28,35,36,47,49]. Overall, simulation-based and procedure-oriented interventions appeared particularly useful for reinforcing technical performance in low-frequency, high-risk pediatric scenarios [9,28,35,47,49].

Non-technical outcomes included self-efficacy, confidence, role clarity, communication, teamwork, leadership, and perceived preparedness. These outcomes were mainly reported after simulation-based, telesimulation, in situ simulation, and interprofessional training interventions [3,9,13,21,28,34,35,50]. Although frequently assessed through self-reported measures, these findings suggest that experiential and team-based educational approaches may support nurses’ perceived readiness and professional confidence in managing pediatric emergencies [13,21,35].

Patient safety and process-related outcomes were less frequently evaluated than knowledge, skills, and confidence. When reported, they included medication reconciliation discrepancies, documentation quality, adherence to care procedures, completion of critical actions, procedural complications, or safety-related indicators [14,22,26,36,37,47,53]. These outcomes suggest a possible link between structured educational interventions and safer clinical processes; however, the limited number of studies assessing patient-level or long-term clinical outcomes prevents firm conclusions [14,22,37,53].

When findings were considered in clinical setting, studies conducted in specialized pediatric environments, such as pediatric intensive care units, neonatal intensive care units, tertiary pediatric hospitals, and pediatric emergency departments, more commonly evaluated high-acuity simulations, procedure-specific competencies, and team-based emergency responses [9,14,19,22,28,33,47,53]. In contrast, studies conducted in general emergency departments, community hospitals, or resource-limited settings more often emphasized baseline pediatric preparedness, feasibility, access to training, confidence, and scalable models such as low-cost simulation, telesimulation, or blended education [5,21,32,35,46,48,50]. This subgrouped narrative synthesis suggests that specialized settings may benefit from advanced simulation targeting complex technical and team-based competencies, whereas general or community settings may particularly benefit from flexible, scalable educational models aimed at improving readiness for low-frequency, high-risk pediatric emergencies.

### 3.7. Non-Technical Skills and Team-Based Learning

Non-technical skills emerged as an important educational target across several innovative training strategies. Simulation-based, in situ simulation, telesimulation, and interprofessional training approaches addressed not only technical performance but also communication, teamwork, leadership, role clarity, situational awareness, and confidence in emergency decision-making [3,9,13,20,21,28,34,50]. Compared with traditional lecture-based or exclusively didactic methods, these strategies provided learners with opportunities to practice team coordination, closed-loop communication, prioritization, and shared decision-making within realistic or semi-realistic pediatric emergency scenarios.

Structured debriefing and personalized feedback were key mechanisms through which innovative educational strategies supported non-technical skill development. Debriefing allowed participants to reflect on team dynamics, decision-making processes, communication errors, and role distribution, while feedback helped identify individual and collective areas for improvement [13,21,28,32]. In situ simulation appeared particularly relevant for non-technical skills because it allowed teams to train within their actual clinical environment, increasing familiarity with local workflows, available resources, and interprofessional interactions [3,9].

Traditional educational approaches, such as lectures or standard in-service training, were useful for consolidating theoretical knowledge and baseline awareness, but they offered fewer opportunities to observe, practice, and correct behavioral and relational components of emergency care. Therefore, the available evidence suggests that innovative and experiential strategies may be particularly valuable for strengthening the soft skills required in pediatric emergencies. However, these outcomes were frequently assessed through self-reported confidence, perceived preparedness, or satisfaction, and only rarely through objective behavioral assessment; therefore, conclusions regarding non-technical skill improvement should be interpreted cautiously.

### 3.8. Contextual and Descriptive Evidence

Descriptive, qualitative, observational, and review-based studies were synthesized separately from the intervention-effectiveness evidence. These studies did not directly evaluate the impact of a defined educational intervention and were therefore not used to infer effectiveness. Instead, they provided contextual information on nurses’ educational needs, baseline knowledge, perceived preparedness, confidence, barriers to implementation, and organizational or resource-related challenges.

Integrative and narrative reviews were included in this contextual synthesis only. They were used to identify broader educational themes, implementation issues, and gaps in the literature, but they did not contribute to the interpretation of intervention effectiveness and were not used to strengthen or confirm findings from primary intervention studies.

Survey and observational studies helped identify areas in which pediatric nurses reported variable knowledge, confidence, or preparedness, including peripheral intravenous catheter management, pediatric delirium, cardiopulmonary resuscitation, intraosseous access, and pediatric emergency readiness [38,41,43]. Qualitative and descriptive studies further highlighted barriers related to workload, limited training opportunities, insufficient organizational support, resource constraints, and context-specific implementation challenges [31,41].

Overall, contextual evidence supported the relevance of continuing education in pediatric emergency nursing by identifying training needs and implementation barriers. However, these findings should be interpreted as background and explanatory evidence rather than as evidence that innovative educational strategies improve knowledge, skills, clinical performance, or patient outcomes.

### 3.9. Methodological Quality and Risk of Bias

The included studies presented several methodological limitations. Many were non-randomized, relied on small or single-center samples, or lacked control groups [4,32]. Cross-sectional studies, while useful for mapping nurses’ knowledge and confidence, were inherently limited by their descriptive design and inability to establish causal relationships [38,43].

Long-term follow-up was infrequently reported, limiting the assessment of skill retention over time [13]. Sampling bias was also common, as convenience sampling was frequently used, raising concerns regarding representativeness and generalizability [31,53]. Additionally, several interventions were conducted in highly specialized tertiary centers, which may not reflect conditions in resource-limited or non-specialist settings [33].

Practical and contextual challenges, including high attrition rates, technological issues during telesimulation, and increased workload or burnout among nurses during the COVID-19 pandemic, further affected participation and completion rates in some studies [21,35].

### 3.10. Narrative Comparison of Intervention Strategies

Among primary intervention studies, simulation-based training appeared to provide the most consistent short-term improvements in studies assessing knowledge, technical performance, and perceived confidence.

However, these findings should be interpreted cautiously because many studies used uncontrolled pre–post designs, small samples, and short-term or self-reported outcomes.

High-fidelity and low-cost simulation workshops were associated with improvements in performance in pediatric life support, trauma care, and management of acute clinical events, with several studies also reporting increased participant satisfaction and confidence [4,13,32].

Blended learning programs appeared useful for integrating theoretical knowledge with practical application, particularly in areas such as safe medication administration and neurological assessment [33,53]. Traditional face-to-face education, while beneficial for reinforcing foundational knowledge, produced more modest outcomes and showed limited evidence of sustained impact. These methods appeared useful for consolidating basic competencies, although the available evidence suggested less consistent effects on skill retention or self-efficacy than interactive and technology-enhanced strategies. [34].

Overall, the available evidence suggests that interactive and technology-supported educational strategies—such as simulation, telesimulation, and blended learning—may offer advantages over purely didactic approaches, particularly when experiential practice and structured feedback are included. However, the strength of this conclusion remains limited by methodological heterogeneity and the scarcity of long-term objective outcomes.

## 4. Discussion

This systematic review synthesized evidence from forty-nine studies published between 2015 and 2025 on innovative continuing education strategies and educational needs in pediatric emergency nursing. As described in the Methods and Results, effectiveness findings were interpreted separately from contextual and descriptive evidence to preserve conceptual clarity. Accordingly, the Results section was structured into two distinct components: effectiveness evidence from primary intervention studies and contextual/descriptive evidence used to interpret educational needs and implementation conditions.

Among the educational strategies identified, simulation-based training appeared to have the most consistent short-term support in the included intervention studies. High-fidelity simulation allows for the replication of complex and emotionally charged clinical scenarios in a controlled and safe environment, facilitating experiential learning and rapid decision-making without jeopardizing patient safety [8,9,21]. The strength of this approach lies in its ability to integrate technical execution with communication, teamwork, and stress management, which are critical components of pediatric emergency care. These findings are consistent with previous literature highlighting simulation as a key driver of improved clinical performance and interprofessional collaboration [7,12].

Among studies evaluating defined educational interventions, blended and digitally supported educational strategies also showed promising outcomes. Programs combining online theoretical modules with in-person simulation or practical sessions were associated with improved coordination among healthcare professionals, enhanced communication, and more efficient emergency responses [8,11,34,43]. These models appear particularly advantageous in balancing flexibility and experiential learning, offering scalable solutions adaptable to different organizational contexts. In contrast, exclusively asynchronous e-learning approaches, while logistically efficient, appeared to provide less direct support for hands-on skills and operational readiness, underscoring the importance of experiential components in emergency training [35,51].

Despite these encouraging findings, the review revealed substantial methodological heterogeneity across studies. Variations in program duration, educational content, sample characteristics, and outcome measures limited direct comparison and precluded quantitative synthesis. A further important limitation concerns the frequent reliance on self-reported outcomes, including perceived competence, confidence, preparedness, and self-efficacy. Although these outcomes are relevant in educational research, they are intrinsically vulnerable to social desirability bias, response-shift bias, and participants’ tendency to report improvement after training exposure. As a result, perceived gains may not correspond to objectively verified improvements in technical performance, non-technical behaviors, clinical decision-making, skill retention, or patient safety outcomes. This limitation may have led to an overestimation of the overall effectiveness of some interventions, particularly in uncontrolled pre–post studies and surveys. Therefore, findings based primarily on self-reported measures should be interpreted as evidence of perceived benefit rather than definitive evidence of improved clinical competence. Future research should combine self-reported outcomes with objective performance assessments, validated behavioral checklists, blinded evaluation where feasible, longitudinal follow-up, and patient- or process-level indicators.

From a clinical and organizational perspective, structured simulation-based continuing education programs may have the potential to support safer pediatric emergency care by improving selected educational, performance-related, and process outcomes, including airway management, cardiopulmonary resuscitation, triage, and medication safety [9,32,53]. However, the extent to which these improvements translate into sustained clinical effectiveness, reduced clinical risk, or improved patient-level outcomes remains uncertain, because few studies assessed objective clinical indicators, long-term practice change, or patient safety endpoints. Improvements in knowledge, confidence, self-efficacy, or simulated performance should therefore not be interpreted as direct evidence of improved patient safety unless supported by objective clinical or patient-level outcomes.

The findings also highlight the importance of integrating non-technical skills training into continuing education programs. As reported in the Results, simulation-based, in situ simulation, telesimulation, and interprofessional approaches appear particularly suited to addressing communication, teamwork, leadership, role clarity, situational awareness, and shared decision-making, because they allow nurses to practice these behaviors within realistic pediatric emergency scenarios. Compared with traditional didactic methods, experiential strategies also provide opportunities for structured debriefing and feedback, which may support reflection on team dynamics and behavioral performance. However, because non-technical outcomes were often measured through self-reported confidence or perceived preparedness, their actual impact on observable team behaviors and clinical outcomes remains uncertain.

This review contributes to the existing literature by highlighting the expanding role of digital and immersive technologies in pediatric nursing education. Emerging tools such as telesimulation and, to a more limited extent, virtual reality (VR), may offer valuable opportunities to train nurses in rare but high-risk clinical scenarios, potentially reducing costs and logistical barriers associated with traditional simulation centers [14,35]. However, in this review, VR was represented by only one included study; therefore, evidence regarding its effectiveness remains preliminary.

Several limitations of this review should be acknowledged. First, although the search strategy was applied consistently across databases and fully reported to ensure reproducibility, it was primarily keyword-based and did not include controlled vocabulary terms, such as MeSH terms or database-specific subject headings. Therefore, relevant studies indexed under alternative terminology may have been missed.

Second, despite protocol registration, PRISMA adherence, and structured methodological appraisal using JBI tools, the included evidence was highly heterogeneous in terms of study design, educational interventions, outcome measures, clinical settings, and follow-up duration. This heterogeneity limited direct comparability across studies, precluded quantitative synthesis, and required a narrative approach. In addition, many studies relied on self-reported outcomes and short-term assessments, while objective measures of clinical performance, skill retention, patient safety, and cost-effectiveness were less frequently reported. These issues may have led to an overestimation of the apparent benefits of some educational strategies and limit the generalizability of the findings to community, non-specialist, or resource-constrained settings.

Third, language restriction and incomplete full-text retrieval may have affected the comprehensiveness of the review. Although only one full-text article was excluded solely because it was published in French, restricting eligibility to English and Italian publications may have introduced language bias and limited the inclusion of evidence from non-English-speaking contexts, including Asian countries or other regions where educational research may be published in local languages. Moreover, nine reports sought for retrieval could not be obtained despite attempts through institutional access, publisher websites, DOI- and title-based searches, Google Scholar, and library holdings. Their unavailability may have resulted in the omission of potentially eligible studies and introduced retrieval bias.

Finally, some methodological refinements were made after the original PROSPERO registration. The registered protocol initially included both undergraduate and post-graduate nursing education, whereas the review was subsequently narrowed to qualified nurses and continuing professional education. This decision improved the clinical coherence and applicability of the synthesis to practicing nurses involved in pediatric emergency care, but may have excluded evidence relevant to pre-licensure nursing education. In addition, integrative and narrative reviews were retained only as secondary contextual evidence. Although their inclusion may have increased methodological heterogeneity and introduced potential overlap with primary studies, secondary sources were not included in the subgrouped effectiveness synthesis, were not used to support or confirm effectiveness claims, and did not influence conclusions regarding the impact of educational interventions.

Future research should prioritize controlled, longitudinal, and multicenter intervention studies using objective performance metrics, patient- or process-level outcomes, extended follow-up periods, and formal cost-effectiveness evaluations. Further studies should also investigate organizational, cultural, and resource-related barriers to support the sustainable implementation of innovative educational strategies across different clinical and economic contexts.

The findings of this review suggest that healthcare institutions should prioritize structured, simulation-based and blended educational programs as part of continuing professional development for pediatric nurses. Educational initiatives should integrate both technical and non-technical skills, be tailored to local organizational contexts, and ensure periodic reinforcement to support skill retention. From a practical perspective, the choice of educational modality should be guided by learning objectives, available resources, staff experience, local risk profiles, and the expected complexity of the clinical scenario. High-fidelity and simulation-based approaches may be particularly valuable for complex pediatric emergencies requiring advanced technical performance, team coordination, and realistic decision-making [9,13,28]. However, these programs may require dedicated simulation facilities, trained faculty, protected training time, equipment, maintenance, and logistical support, which may limit feasibility and sustainability in community hospitals, general emergency departments, and resource-limited settings.

In such contexts, scalable and lower-cost strategies may represent more sustainable alternatives. Low-cost simulation, in situ simulation, blended learning, and telesimulation can support pediatric emergency preparedness while reducing infrastructure and travel-related barriers [5,21,32,35,48,50]. Low-cost simulation may rely on basic mannequins, locally available equipment, standardized scenarios, and structured debriefing, whereas in situ simulation allows teams to train within their actual clinical environment and become familiar with local workflows, equipment, and role distribution [3,9,32]. Telesimulation and blended approaches may further expand access to training by allowing theoretical content, remote facilitation, and expert feedback to be delivered across geographically dispersed or underserved settings [21,35]. However, formal cost-effectiveness evidence remains limited, as few studies assessed implementation costs, cost per learner, faculty time, equipment maintenance, or downstream clinical and patient-safety outcomes. Therefore, high-fidelity simulation should not be considered inherently superior or universally necessary; rather, a tiered approach may be preferable, combining online or blended modules for theoretical knowledge, low-cost or in situ simulation for core emergency skills and teamwork, telesimulation for remote support, and selective use of high-fidelity simulation for rare, complex, or high-risk scenarios.

## 5. Conclusions

Overall, evidence from primary intervention studies suggests that simulation-based, blended, and telesimulation-based educational strategies may be associated with short-term improvements in nurses’ knowledge, technical performance, self-efficacy, and selected non-technical skills in pediatric emergency care. However, these findings should be interpreted cautiously, as much of the available evidence derives from non-controlled designs, small samples, short-term assessments, simulated performance measures, or self-reported outcomes. Evidence on virtual reality was limited to one included study and should therefore be considered preliminary.

Contextual and descriptive studies highlighted persistent educational needs, perceived barriers, preparedness, and implementation factors, but were not used to infer intervention effectiveness. From a practical perspective, scalable approaches such as low-cost simulation, in situ simulation, blended learning, and telesimulation may help support pediatric emergency preparedness in resource-limited settings, although formal cost-effectiveness evidence remains limited.

Future research should prioritize robust, longitudinal, and multicenter intervention studies incorporating objective performance measures, patient- or process-level outcomes, longer follow-up, and formal cost-effectiveness evaluations. Stronger evidence is needed before firm conclusions can be drawn regarding the impact of advanced and experiential continuing education on clinical competence, sustained clinical effectiveness, clinical risk reduction, and patient safety.

## Figures and Tables

**Table 1:** Search strategies by database.

Database	Search Strategy	Limits Applied
PubMed	(nurs AND (“continuing education” OR “nursing education” OR “professional development”) AND (“pediatric emergency” OR “emergency pediatric nursing” OR “child emergency care”) AND (effectiveness OR “treatment outcome” OR “nursing knowledge” OR “nurse competency”))	January 2015–August 2025; English or Italian
CINAHL Complete	(nurs AND (“continuing education” OR “nursing education” OR “professional development”) AND (“pediatric emergency” OR “emergency pediatric nursing” OR “child emergency care”) AND (effectiveness OR “treatment outcome” OR “nursing knowledge” OR “nurse competency”))	January 2015–August 2025; English or Italian
Scopus	(nurs AND (“continuing education” OR “nursing education” OR “professional development”) AND (“pediatric emergency” OR “emergency pediatric nursing” OR “child emergency care”) AND (effectiveness OR “treatment outcome” OR “nursing knowledge” OR “nurse competency”))	January 2015–August 2025; English or Italian
Cochrane Library	(nurs AND (“continuing education” OR “nursing education” OR “professional development”) AND (“pediatric emergency” OR “emergency pediatric nursing” OR “child emergency care”) AND (effectiveness OR “treatment outcome” OR “nursing knowledge” OR “nurse competency”))	January 2015–August 2025; English or Italian

**Table 2:** Methodological appraisal and quality assessment of included studies.

1. Randomized controlled trials (RCTs)—n = 3.					
**Author (Year)**	**Short Title**	**Study Design**	**JBI Checklist**	**Main Critical Domains**	**Overall Judgment**
Polat et al., 2025 [16]	Adhesive remover & stress ball	RCT	JBI RCT	Blinding not feasible; subjective pain and fear outcomes; limited age range; appropriate statistical analysis	Moderate
Abbass et al. (2023) [17]	ADDIE-based pediatric transfusion program	RCT	JBI RCT	Cluster randomization insufficiently described; observational outcome measures; acceptable internal validity	Moderate
Pillai Riddell et al. (2023) [18]	“Shot Blocker” for venipuncture	RCT	JBI RCT	Blinding not feasible; partially subjective outcomes; appropriate statistical analysis	Moderate
2. Quasi-experimental studies (pre–post with or without control group)—n = 25.					
**Author (Year)**	**Short Title**	**Study Design**	**JBI Checklist**	**Main Critical Domains**	**Overall Judgment**
LaRosa (2025) [19]	Early mobility curriculum in PICU	Pre–post (simulation-based)	JBI Quasi-experimental	Small sample size; no control group; OSCE and self-efficacy outcomes	Moderate
Park et al. (2024) [20]	Self-learning vs. instructor-led pediatric CPR	Quasi-RCT	JBI Quasi-experimental	Non-random allocation; partially objective outcomes	Moderate
Montgomery et al. (2021) [21]	Telesimulation for community ED nurses	Pre–post	JBI Quasi-experimental	Limited sample; self-selection bias; perceptual outcomes	Moderate
Gupta et al. (2019) [3]	Interprofessional resuscitation program	Pre–post	JBI Quasi-experimental	Small sample; subjective outcomes; uncontrolled confounders	Moderate–High
Dalesio et al. (2019) [22]	Multidisciplinary pediatric airway program	Descriptive series	JBI Quasi/Case series	No control group; descriptive outcomes	High
Chen et al. (2019) [5]	Pediatric emergency curriculum in Tanzania	Quasi-experimental with control	JBI Quasi-experimental	Non-random matching; knowledge-based outcomes	Moderate
Ibrahim & Yahya (2023) [23]	Dehydration management in children < 5 years	Quasi-experimental with control	JBI Quasi-experimental	Groups from different hospitals; acceptable internal validity	Moderate
Hazwani et al. (2020) [9]	Pediatric resuscitation simulation (“Mock Code”)	Pre–post	JBI Quasi-experimental	Observational outcomes; potential observer bias	Moderate
Ahmed et al. (2021) [24]	Multimedia pediatric tracheostomy module	Pre–post	JBI Quasi-experimental	Knowledge and confidence outcomes; no control group; short-term evaluation; self-report measures	Moderate
Baran & Turan (2018) [25]	Pediatric emergency training (Turkey)	Pre–post	JBI Quasi-experimental	Small sample; lack of follow-up	Moderate–Low
Alruwaili et al. (2025) [14]	Virtual reality (VR) simulation for neonatal emergency training	Pre–post	JBI Quasi-experimental	No randomized allocation; perceptual and performance outcomes; single-site; limited long-term follow-up	Moderate
Cooper et al. (2024) [26]	ESCALATION system simulation education	Pre–post	JBI Quasi-experimental	Mixed-methods; simulation-based outcomes; limited follow-up; no randomization	Moderate
Downey et al. (2023) [27]	Port access education innovation	Pre–post	JBI Quasi-experimental	Process outcomes; no control group; descriptive improvement data	Moderate
Gilfoyle et al. (2017) [28]	Interprofessional resuscitation simulation	Pre–post	JBI Quasi-experimental	No control group; performance-based outcomes; potential observer bias	Moderate–High
Lee et al. (2019) [29]	Algorithm-based dehydration management	Pre–post	JBI Quasi-experimental	Self-reported measures; confounding risk	Moderate
Behzadi et al. (2019) [30]	Oral care for ventilated children	Pre–post	JBI Quasi-experimental	Observational outcomes; no control group	Moderate–High
Mitchell et al. (2020) [13]	Simulation for managing aggression	Pre–post	JBI Quasi-experimental	Perceptual outcomes; absence of clinical endpoints	Moderate–High
Kusi Amponsah et al. (2020) [31]	Barriers to pediatric pain management (Ghana)	Pre–post	JBI Quasi-experimental	Context-specific findings; no intervention comparison; transferability limits	Moderate–High
Lima et al. (2023) [32]	Low-cost pediatric trauma workshop	Pre–post	JBI Quasi-experimental	Short-term evaluation; no follow-up; knowledge/skills outcomes	Moderate–High
Loutfy et al. (2023) [33]	Level of consciousness education program	Pre–post	JBI Quasi-experimental	Non-random allocation; observational performance assessment	Moderate
Paek et al. (2019) [34]	Child abuse education pilot study	Pre–post	JBI Quasi-experimental	Small pilot sample; self-report outcomes; no control group	Moderate
Vestergaard et al. (2017) [4]	Self-led vs. instructor-led pediatric BLS	Pre–post	JBI Quasi-experimental	Non-randomized allocation; skill-based outcomes; potential performance bias	Moderate
Thomas et al. (2023) [35]	Emergency distance-learning curriculum	Pre–post	JBI Quasi-experimental	Educational outcomes only; limited generalizability	Moderate
Yabrodi et al. (2025) [36]	Educational game for central venous catheter care	Pre–post	JBI Quasi-experimental	No clinical outcomes; simulated performance only	Moderate
Shivani & Joseph (2022) [37]	SPC care reinforcement program	Pre–post	JBI Quasi-experimental	No control group; knowledge/adherence outcomes; short-term evaluation; potential Hawthorne effect	Moderate
3. Cross-sectional observational studies (surveys, knowledge, attitudes)—n = 11.					
**Author (Year)**	**Short Title**	**Study Design**	**JBI Checklist**	**Main Critical Domains**	**Overall Judgment**
Indarwati et al. (2022) [38]	Pediatric PIVC knowledge/confidence	Multicenter cross-sectional	JBI Cross-sectional	Self-selection; social desirability bias	Moderate–High
Cicolini et al. (2023) [39]	Intraosseous access	Cross-sectional	JBI Cross-sectional	Convenience sample; structured tool	Moderate
Cho et al. (2020) [40]	SUID and pediatric resuscitation	Cross-sectional	JBI Cross-sectional	Knowledge outcomes; local context	Moderate
Abdel Razeq et al. (2025) [41]	Barriers in SCD pain management	Cross-sectional	JBI Cross-sectional	Self-report; limited representativeness	High
Hill et al. (2025) [42]	Competency and continuing education	Cross-sectional	JBI Cross-sectional	Non-causal analysis; uncontrolled confounders	Moderate–High
Ercan & Kızıler (2023) [43]	Pediatric ICU delirium	Cross-sectional	JBI Cross-sectional	Low awareness of tools; social desirability bias	Moderate
Sim et al. (2021) [44]	Pediatric emergency care in Armenia	Cross-sectional	JBI Cross-sectional	Convenience sampling; confounding factors	Moderate–High
Hendy et al. (2023) [45]	CPR self-efficacy and stress	Cross-sectional	JBI Cross-sectional	Self-report; large sample; non-causal	Moderate–High
McNeill (2016) [46]	Self-efficacy in pediatric emergencies	Correlational	JBI Cross-sectional	Small sample; informative for training gaps	High
Başer & Anil, (2025) [6]	Pediatric Emergency Course	Cross-sectional	JBI Cross-sectional	Perceived self-efficacy; self-assessment	Moderate
Cicolini et al. (2023) [39]	SUID and infant CPR knowledge	Cross-sectional	JBI Cross-sectional	Self-report questionnaire; single-country context; limited external validity	Moderate
4. Time-series, applied cohort, and quality/implementation studies—n = 7.					
**Author (Year)**	**Short Title**	**Study Design**	**JBI Checklist**	**Main Critical Domains**	**Overall Judgment**
Blick et al. (2021) [47]	Ultrasound-guided PIVC	Consecutive post-training series	JBI Case series/Cohort	No control group; objective outcomes	Moderate
Salehi et al. (2021) [48]	Scaling-up pediatric nurse education (Ghana)	Longitudinal mixed-methods	JBI Cohort/Mixed	OSCE at 14 months; mentoring variability	Moderate
Good et al. (2019) [49]	US-guided procedural motion study	Technical study	JBI Diagnostic	Objective metrics; limited transferability	Moderate
Nader et al. (2024) [50]	Distance-learning empowerment (Ghana)	Mixed-methods	JBI Cohort/Mixed	Perceptual outcomes; single center	Moderate–Low
Arrojo & Hooshmand, (2021) [51]	Depression screening in pediatric ED	QI time-series	JBI Cohort/Case series	Process outcomes; possible co-interventions	Moderate
Kersaint Edme et al. (2025) [52]	Caregiver asthma management	QI pre–post	JBI Cohort/Quality	Process measures; uncontrolled confounders	Moderate–High
McDonald et al. (2018) [53]	Nursing process optimization	Quality improvement series	JBI Quality	Multiple confounders; process outcomes	Moderate
5. Integrative and narrative reviews—n = 3.					
**Author (Year)**	**Short Title**	**Study Type**	**Quality Tool**	**Main Critical Domains**	**Overall Judgment**
Amponsah et al. (2019) [54]	Educational strategies for pediatric pain	Integrative review	Whittemore–Knafl	Knowledge/attitude improvements; limited clinical outcomes	Moderate
Cho et al. (2020) [55]	Analgesia interventions in hospitalized children	Integrative review	Qualitative appraisal	High heterogeneity; publication bias risk	Moderate
Recznik, C.T. & Simko, L.M. (2018) [56]	Pediatric triage education	Integrative review	Whittemore–Knafl	High heterogeneity; no PRISMA; favorable signal for simulation	Moderate

**Table 3:** Characteristics of included studies and educational interventions (n = 49).

Author (Year)	Study Design	Educational Intervention	Participants	Key Outcomes	Follow-Up/Long-Term Impact	Main Limitations
Vestergaard et al. (2017) [4]	Prospective controlled quasi-experimental (partial randomization)	Self-directed online PBLS video + mannequin vs. 2 h instructor-led course (ERC 2005)	58 nurses (pediatric + maternity units; university hospital)	Practical simulated pediatric arrest performance; self-confidence/willingness: no between-group difference (*p* = 0.51)	Not assessed	Partial randomization; different timing/material access; different mannequins; no skill retention/non-inferiority evaluation
McDonald et al. (2018) [53]	Single-group quasi-experimental pre–post	Structured pharmacist-led education on medication reconciliation (errors, standardized questions, checklist)	Pediatric ED setting; nurses/pharmacists involved; 200 pediatric patients evaluated	↓ discrepancies per patient (4 → 2; *p* = 0.0023) and per drug (3 → 1.5; *p* = 0.0002); improved documentation	Not assessed	Clinical harm not measured; convenience sampling; potential documentation/recall bias
Thomas et al. (2023) [35]	Pilot single-group quasi-experimental pre–post	Telesimulation + weekly asynchronous micro-learning (modules, podcasts, skill demos) over 12 weeks	110 emergency nurses (USA/Canada)	Knowledge median 70 → 88 (*p* = 0.018); critical actions checklist 60 → 100 (*p* = 0.016)	Not assessed	Selection bias; low completion; technology barriers; no real patient outcomes
Loutfy et al. (2023) [33]	Single-group quasi-experimental pre–post (two sites)	Structured teaching program (lectures, small-group discussions, demonstrations, supervised practice) over 10 weeks	49 PICU nurses (tertiary hospitals)	↑ knowledge, practice, self-confidence on LOC scales (*p* < 0.001)	Not assessed	Small sample; two centers; no control group; limited generalizability
Arrojo et al. (2021) [51]	Pilot study	In-service education for staff + individual education for families on adolescent depression screening/resources	20 pediatric ED staff (mixed roles)	↑ knowledge (pre–post; *p* < 0.001)	Not assessed	Rural small population; follow-up dependent on families; limited contact/traceability
Lima et al. (2023) [32]	Single-group quasi-experimental pre–post	Low-cost pediatric trauma workshop (theory + practice) 5 days/13 h	20 participants (35% nursing students; 65% nurse residents)	↑ knowledge; satisfaction and self-esteem in learning improved	Not assessed	Very small single-site sample; no randomization; limited generalizability
Montgomery et al. (2021) [21]	Multicenter descriptive pilot	Pediatric telesimulation via Zoom + SimBox (status epilepticus stabilization)	86 nurses (community EDs; USA/Canada)	Post-session perceived gains (critical actions, meds list, transfer needs, confidence); high satisfaction measures used (SET-M/NPS)	Not assessed	COVID workload/burnout; tech/connectivity issues; participation variability; no follow-up/patient outcomes
Indarwati et al. (2022) [38]	Multicenter cross-sectional	Not an intervention (survey study)	413 pediatric nurses (Indonesia; multiple hospital types/units)	Knowledge and confidence in PIVC insertion/maintenance; training/experience associated with higher scores (*p* < 0.05)	Not applicable	Limited predictors considered; omitted leadership/resources/environment factors
Abdel Razeq et al. (2025) [41]	Descriptive cross-sectional	Not an intervention (barriers survey)	298 nurses (Jordan; pediatric units/PICU/ED/hematology)	Perceived barriers to optimal SCD pain management (workload, time, support, training)	Not applicable	Convenience sampling; setting/geography may limit generalizability
Kusi Amponsah et al. (2020) [31]	Descriptive qualitative	Not an intervention (qualitative barriers)	28 pediatric nurses (Ghana; varied hospital types)	Nursing-related barriers to pediatric pain management (qualitative themes)	Not applicable	Transferability only; other stakeholders not included; facilitators not explored
Ercan et al. (2023) [43]	Descriptive	Not an intervention (knowledge/attitude survey)	80 ICU nurses (Turkey; tertiary ICUs)	Pediatric delirium knowledge/attitudes (questionnaire scoring 0–60)	Not applicable	Single-site/region; limited generalizability
Mitchell et al. (2020) [13]	Pilot survey with quasi-experimental elements	2 h simulation-based training for managing clinical aggression (MOCA day)	146 staff (83% nursing; tertiary pediatric hospital)	↑ perceived confidence/competence; maintained at 3–6 months (*p* < 0.01)	Yes (3–6 months)	Low follow-up response; anonymity prevented linking; Kirkpatrick levels 1–2 focus
Chen et al. (2019) [5]	Pilot quasi-experimental (non-randomized)	2.5-day pediatric emergency curriculum (lectures, skills stations, simulations)	ED nurses (Tanzania); intervention n = 15 (11 complete), control n = 14 (11 complete)	↑ knowledge/self-efficacy post-training; behavior checklist after training	Yes (7 weeks)	Small sample; single site; non-randomized; limited action metrics; observer reliability unclear
Cicolini et al. (2023) [39]	Integrative review	Review of pediatric pain alleviation interventions for hospitalized childre	Varied pediatric inpatient populations and pediatric nurses across included studies	Effective pharmacological and non-pharmacological pain strategies; improved pain-related outcomes; emphasized nurses’ competence and recognition of pediatric pain; highlighted persistent under-management of pediatric pain	Not assessed	Heterogeneous interventions; no long-term outcomes
Ahmed et al. (2021) [24]	Prospective observational	Online multimedia pediatric tracheostomy care module	Mixed providers; nurses 66%; large completion numbers (module n = 422)	↓ lack of confidence (*p* < 0.001); ↑ knowledge quiz (83.0% → 88.6%; *p* < 0.00001)	Not assessed	No gold standard; voluntary response bias; same quiz items pre/post; limited verification
Yabrodi et al. (2025) [36]	Pilot single-arm pre–post	Serious game + 15 min recorded presentation (central line care)	32 PICU/cardiac ICU nurses	↑ simulated performance; improvements in hand hygiene/site prep/placement (*p* ≤ 0.04); overall performance improved (*p* < 0.01)	Yes (90 days considered)	No control group; limited engagement metrics; single institution; sustainability/patient outcomes not assessed
Alruwaili et al. (2025) [14]	Mixed-methods with quasi-experimental elements	Virtual reality (VR)-based neonatal emergency simulation program (2 weeks; 3 sessions; real-time feedback)	128 NICU nurses (4 hospitals)	↑ OSCE, MCQ retention, decision accuracy; ↓ stabilization time; ↓ safety events (all *p* < 0.001)	Limited (short program; no true long-term)	Non-random allocation; short follow-up; tech issues (cybersickness); generalizability limits
Paek et al. (2019) [34]	Pilot survey study	Two 2 h structured lectures on child abuse assessment/reporting	663 completers (nurses 61.7% + others; Korea)	↑ knowledge, confidence, willingness to report (*p* < 0.001)	Yes (3 months)	Limited to ED-focused providers; adherence differences; missing nonparticipant data; short study
McNeill (2016) [46]	Correlational (with workshop described)	3-day structured workshop (presentations, simulations, debriefing, pre/post tests)	37 nurses (community hospital; pediatric ED/outpatient)	↑ knowledge and self-efficacy (pre/post comparisons)	Not assessed	Real clinical practice hard to assess (rare events); small sample; no interdisciplinary participation
Gilfoyle et al. (2017) [28]	Single-group quasi-experimental pre–post	Team-based resuscitation simulation training (Teams4Kids; lecture + video + multiple scenarios)	300 participants (incl. 43.3% nurses; Canada)	↑ CPT performance; ↓ time to compressions/defibrillation (*p* ≤ 0.001)	Not assessed	Simulation-only outcomes; no RCT; components not isolated; no retention assessment
Cicolini et al. (2023) [39]	Multicenter cross-sectional	Not an intervention (knowledge/experience survey on IO access)	432 nurses (Italy; multiple departments)	Knowledge of IO guidelines and self-reported experience	Not applicable	Convenience sample; self-report bias; protocols/training recency unknown; pediatric nurses underrepresented
Gupta et al. (2019) [3]	Pilot survey	Interprofessional, small-group, fully in situ simulation sessions (1 h; 16 sessions/4 months)	37 inpatient providers (mixed roles; pediatrics)	Perceived usefulness (post-session questionnaires)	Not assessed	Limited scale; recruitment/scheduling barriers; no objective learning/patient outcomes
Hendy et al. (2023) [45]	Descriptive cross-sectional	Not clearly specified (training exposure not detailed)	748 pediatric nurses (6 hospitals)	Self-assessed CPR capabilities, stress, attitudes; associations with factors (*p* < 0.05)	Not applicable	No qualitative component; single timepoint
Good et al. (2019) [49]	Prospective cohort with pre–post	3-phase US-guided PIV program (online module + 2 h simulation + supervised practice)	27 (21 nurses, 6 experts)	Improved hand motion metrics and procedure time (*p* < 0.0001); clinical attempts tracked	Yes (4 months; attempts/success)	No a priori sample size; simulation setting; expert blinding issues; self-reported clinical attempts
Kersaint Edme et al. (2025) [52]	QI initiative (post-test descriptive)	Culturally tailored asthma action plan education + video via QR/EHR	Pediatric pulm/allergy staff (n = 32; nurses + physicians)	Utilization (YouTube views) and staff satisfaction	Not assessed	No caregiver outcomes; low response; limited participant demographics; usage metric limitations
LaRosa et al. (2025) [19]	Single-group quasi-experimental pre–post	Simulation-based early mobility curriculum (HF scenarios + debrief + short didactic)	11 PICU nurses	↑ knowledge, OSCE competence, self-efficacy (*p* ≤ 0.031)	Not assessed	Very small sample; no randomization; potential rater relationship bias; no bedside outcomes
Cooper et al. (2024) [26]	Mixed methods, quasi-experimental	Online ESCALATION package + clinical simulation scenarios	14 participants (78.6% nurses)	High self-reported preparedness; documentation completeness; communication quality; family involvement	Not assessed	Small sample; response bias; limited authenticity for clinical outcomes; limited validation of new measures
Recznik et al. (2018) [56]	Integrative review	Multiple pediatric triage education approaches (ETAT/ETAT+, CTAS, ESI, JumpSTART, etc.)	Varied healthcare providers; ED/triage contexts	Generally ↑ triage knowledge/accuracy/self-efficacy; some retention decline over time	Variable (3–9 months in included studies)	Heterogeneity; retrospective audits/surrogates; design/implementation underreported; language limits
Sim et al. (2021) [44]	Cross-sectional survey	Not an intervention (knowledge/attitudes survey)	175 out-of-hospital emergency nurses (ambulance services)	Knowledge/attitudes on pediatric emergency care (survey outcomes)	Not applicable	Limited by survey design; intervention details not applicable
Dalesio et al. (2019) [22]	Descriptive	Multidisciplinary pediatric airway program (1-day CME; semiannual; didactic + skills + simulation)	Multidisciplinary team (incl. specialized nurses; tertiary pediatric hospital)	Reported reductions in airway-related morbidity/calls; improved first-pass success (as described)	Yes (program longitudinal 2008–2018)	Requires 24/7 pediatric expertise; resource/equipment constraints; measurement details not fully specified
Pillai Riddell et al. (2023) [18]	Randomized controlled trial (as reported)	Nurse-targeted educational strategies in pain context (details limited in extraction)	228 school-aged children (pain outcomes)	Wong-Baker pain scores significantly lower in intervention group (*p* < 0.001)	Not assessed	Product availability/context limits; setting-specific generalizability; implementation constraints
Amponsah et al. (2019) [54]	Integrative review	Educational strategies for pediatric pain assessment/management (interactive teaching, brochures, lectures)	Samples varied; nurses > 1/3 in included studies	Improvements in knowledge/attitudes/practice reported across studies	Not clearly reported	Grey literature excluded; language limits; heterogeneity across studies
Cho et al. (2020) [40]	Descriptive cross-sectional	Not an intervention (knowledge/confidence survey)	136 pediatric nurses (Korea; multiple units)	Knowledge on safe sleep and infant CPR; confidence via VAS	Not applicable	Clinical-only sample; limited setting representativeness; new tools with limited validation
Baran et al. (2018) [25]	Single-group quasi-experimental pre–post	Educational booklet + in-person training on fever/febrile seizures	126 pediatric nurses (Turkey)	↑ knowledge score (*p* = 0.000)	Yes (~4 weeks)	No control; single site; incomplete post-tests; limited generalizability
Downey et al. (2023) [27]	Descriptive pilot	Simulation-based port access curriculum using wearable trainer (10 sessions; 2 h)	34 pediatric ED nurses (PEN)	↑ knowledge and self-efficacy; knowledge maintained at 3 months	Yes (3 months)	Single center; small all-female sample; limited settings/age range; resource replication concerns
Nader et al. (2024) [50]	Pilot longitudinal mixed methods	Distance curriculum + interactive sessions + simulation (10 months; biweekly)	25 PICU nurses engaged; 15 in simulations (Ghana)	Team performance (CTS) and perceived simulation effectiveness (SET-M) improved	Not assessed	Inconsistent attendance; rater variability early; self-report/team-simulation focus only
Hill et al. (2025) [42]	Retrospective descriptive subanalysis	Not an intervention (associations: competency/certification/CE vs. readiness)	3557 EDs (system-level; nurses majority)	Associations with NPRP weighted pediatric readiness score (WPRS)	Not assessed	Self-reported site data; unverifiable responses; missing data exclusions; terminology ambiguity
Blick et al. (2021) [47]	Descriptive	Structured US-guided PIV insertion training (30 min video + 2–4 h practice)	83 pediatric ED nurses	First-attempt success improved (67% → 83% by 10th encounter); complications ~25%	Not assessed	Retrospective data; confounding experience factors; EMR complications incomplete; no long-term impact
Park et al. (2024) [20]	Prospective RCT	Self-learning video vs. instructor-assisted pediatric CPR training + feedback manikin	97 nurses (general hospital)	↑ self-efficacy post; decline at 1 year; CPR performance declined at 1 year	Yes (1 year)	Single center; high loss to follow-up; no pre-training performance baseline
Salehi et al. (2021) [48]	Mixed methods quasi-experimental pre–post	1-year pediatric nurse specialist education (interactive teaching, cases, simulation, experiential)	330 graduates (Ghana)	↑ knowledge; ↑ confidence; OSCE competence maintained	Yes (14 months)	OSCE reliability limits; limited baseline data; incomplete follow-up across cohorts
Hazwani et al. (2020) [9]	Retrospective descriptive	In situ mock code simulation program (2–3/month) + structured debrief	Multidisciplinary PICU/ED teams (Saudi Arabia)	Improved leader and team performance scores; faster key actions over 6 months	Yes (6 months)	No pre-training CPR knowledge measures; simulation-only outcomes; no patient outcomes
Abbass et al. (2023) [17]	Randomized clinical trial (pre–post)	ADDIE-based training on pediatric blood transfusion (3 × ~2 h over 2 weeks)	60 pediatric nurses (Iraq)	Performance checklist score markedly improved (*p* < 0.001)	Not assessed	Single observer checklist (mitigated by repeated observations); no long-term follow-up
Cho et al. (2020) [55]	Integrative review	Not a single intervention (pain alleviation interventions literature)	Varied; pediatric nursing contexts	Summarized physiological/behavioral pain outcomes across interventions	Not applicable	Heterogeneity; pain under-recognition issues; not focused on retention/implementation
Shivani et al. (2022) [37]	Quasi-experimental pre–post	Reinforcement program on short peripheral catheter (SPC) care (individual + group sessions)	44 pediatric ward/PICU nurses (India)	↑ guideline adherence (*p* = 0.001); ↓ early phlebitis (66.7% → 37.5%; *p* = 0.027)	Not assessed	Limited observations; no practice follow-up; workload/resources not controlled
Behzadi et al. (2019) [30]	Quasi-experimental pre–post	Structured oral care education (lectures, Q&A, practice, videos)	100 PICU nurses (Tehran)	Performance score improved (*p* < 0.001); increased chlorhexidine/toothbrush use	Yes (4 weeks)	Hawthorne effect risk; short duration; convenience sampling; need ongoing feedback
Lee et al. (2019) [29]	Quasi-experimental pre–post	Algorithm-based epilepsy care education (60 min; lecture + practice)	27 nurses (peds neuro + pediatric ED)	↑ knowledge and self-efficacy; maintained at 1 week	Yes (1 week)	Single site; no control group
Ibrahim et al. (2023) [23]	Quasi-experimental pre–post	(Details limited) dehydration management knowledge training	48 pediatric nurses (Kurdistan region)	↑ knowledge scores (*p* < 0.0001); slight decline at post-test 2	Yes (post-test 2; timing not fully specified)	Control group knowledge increased; demographic correlations may limit generalizability; training exposure variable
Başer et al. (2025) [6]	Quasi-experimental pre–post	2-day pediatric emergency CPD course (interactive/participatory; ARCS model)	57 nurses	Large knowledge gains (*p* < 0.001); motivation/confidence/satisfaction improvements	Not assessed	Sample adequacy issues (KMO); some reliability below ideal; no long-term follow-up
Polat et al. (2025) [16]	Parallel-group RCT	Procedural pain/fear intervention (adhesive remover spray + stress ball)	Pediatric patients (6–9 years)	↓ pain and fear vs. control (*p* < 0.001)	Not assessed	Limited age range; no physiological/hormonal outcomes; not generalizable to chronic/neurologic conditions

The upward and downward arrows indicate an increase and a decrease, respectively.

## Data Availability

No new data were created or analyzed in this study. Data sharing is not applicable to this article.

## Supplementary Materials

The following supporting information can be downloaded at: https://www.mdpi.com/article/10.3390/pediatric18030080/s1, Table S1: PRISMA 2020 Checklist [57].

## Abbreviations

PBLS, Pediatric Basic Life Support; PALS, Pediatric Advanced Life Support; CPR, cardiopulmonary resuscitation; ED, Emergency Department; PICU, Pediatric Intensive Care Unit; NICU, Neonatal Intensive Care Unit; OSCE, Objective Structured Clinical Examination; SET-M, Modified Simulation Effectiveness Tool; CTS, Clinical Teamwork Scale; NPS, Net Promoter Score; WPRS, Weighted Pediatric Readiness Score; NPRP, National Pediatric Readiness Project; PIVC, Peripheral Intravenous Catheter; IO, Intraosseous; US-guided PIV, ultrasound-guided peripheral intravenous catheter; VR, Virtual Reality; QI, Quality Improvement; RCT, randomized controlled trial; HF, high-fidelity; LOC, level of consciousness; GCS, Glasgow Coma Scale; PGCS, Pediatric Glasgow Coma Scale; PFSS, Pediatric Full Outline of UnResponsiveness Score; SPC, short peripheral catheter; VIP, Visual Infusion Phlebitis scale; SCD, sickle cell disease; CE, continuing education; CME, continuing medical education.
